# Bisdemethoxycurcumin Protects Small Intestine from Lipopolysaccharide-Induced Mitochondrial Dysfunction via Activating Mitochondrial Antioxidant Systems and Mitochondrial Biogenesis in Broiler Chickens

**DOI:** 10.1155/2021/9927864

**Published:** 2021-11-09

**Authors:** Jingfei Zhang, Yuxiang Yang, Hongli Han, Lili Zhang, Tian Wang

**Affiliations:** ^1^College of Animal Science and Technology, Nanjing Agricultural University, Nanjing 210095, China; ^2^Bluestar Adisseo Nanjing Co., Ltd., Nanjing 210047, China

## Abstract

Bisdemethoxycurcumin is one of the three curcuminoids of turmeric and exhibits good antioxidant activity in animal models. This study is aimed at investigating the effect of bisdemethoxycurcumin on small intestinal mitochondrial dysfunction in lipopolysaccharide- (LPS-) treated broilers, especially on the mitochondrial thioredoxin 2 system and mitochondrial biogenesis. A total of 320 broiler chickens were randomly assigned into four experimental diets using a 2 × 2 factorial arrangement with diet (0 and 150 mg/kg bisdemethoxycurcumin supplementation) and stress (saline or LPS challenge) for 20 days. Broilers received a dose of LPS (1 mg/kg body weight) or sterile saline intraperitoneally on days 16, 18, and 20 of the trial. Bisdemethoxycurcumin mitigated the mitochondrial dysfunction of jejunum and ileum induced by LPS, as evident by the reduced reactive oxygen species levels and the increased mitochondrial membrane potential. Bisdemethoxycurcumin partially reversed the decrease in the mitochondrial DNA copy number and the depletion of ATP levels. Bisdemethoxycurcumin activated the mitochondrial antioxidant response, including the prevention of lipid peroxidation, enhancement of manganese superoxide dismutase activity, and the upregulation of the mitochondrial glutaredoxin 5 and thioredoxin 2 system. The enhanced mitochondrial respiratory complex activities in jejunum and ileum were also attributed to bisdemethoxycurcumin treatment. In addition, bisdemethoxycurcumin induced mitochondrial biogenesis via transcriptional regulation of proliferator-activated receptor-gamma coactivator-1alpha pathway. In conclusion, our results demonstrated the potential of bisdemethoxycurcumin to attenuate small intestinal mitochondrial dysfunction, which might be mediated via activating the mitochondrial antioxidant system and mitochondrial biogenesis in LPS-treated broilers.

## 1. Introduction

Chickens suffer from a high prevalence of small intestinal mucosal injury, which poses a major threat to the health of poultry [1]. Oxidative damage and the subsequent mitochondrial dysfunction are common characteristic features of small intestinal mucosal injury. The biomarkers of mitochondrial dysfunction include mitochondrial integrity, mitochondrial membrane potential, mitochondrial DNA (mtDNA) content, mitochondrial antioxidant defense system, mitochondrial respiratory complex activities, and mitochondrial biogenesis [2, 3]. Among them, the inactivation of the mitochondrial redox system induced by excessive reactive oxygen species (ROS) acts causally in mitochondrial dysfunction. Under a sustained high ROS level, nonenzymatic antioxidants are depleted, and antioxidant enzymatic activities are inhibited, resulting in the disruption of redox balance within mitochondria [4]. Several antioxidant and redox systems, such as the glutathione (GSH)-glutaredoxin (Grx) and thioredoxin (Trx) system, participate in ROS elimination and maintaining mitochondrial function in a redox-dependent manner [5]. The Trx2 system, comprising Trx2, thioredoxin reductase 2 (TrxR2), and peroxiredoxin 3 (Prx3), catalyzes the reductive metabolism of peroxides specifically targeted in mitochondrial fraction [6, 7]. Moreover, the Trx2 system plays an important role in maintaining mitochondrial proteins in their reduced state, which is essential for the modification of mitochondrial function [8].

Mitochondrial biogenesis is a complex progress that mediates the proliferation of healthy mitochondria and clearance of impaired mitochondria. Accumulation of defective mitochondrial and decreasing mtDNA is referred to as direct contributors to mitochondrial dysfunction and breakdown [9, 10]. Proliferator-activated receptor-gamma coactivator-1alpha (PGC-1*α*) is a cotranscriptional regulation factor that induces mitochondrial biogenesis by activating nuclear respiratory factor 1 and 2 (NRF1 and NRF2) and subsequently mitochondrial transcription factor A (TFAM) [11, 12]. At the transcriptional level, PGC-1*α* signaling coordinates the machinery leading to increased mitochondrial mtDNA, reduced electron leak, and decreased ROS production, thus allowing mitochondria reconnection to antioxidant response and preventing the consequences of mitochondrial dysfunction [13]. Therefore, PCG-1*α* signaling, along with NRF and TRAF, is deemed to be an important target in mtDNA repair and improvement of mitochondrial function.

The animal model of lipopolysaccharide- (LPS-) induced immunological and oxidative stress is widely used in antioxidant and mitochondrial damage research. In the LPS model, a burst of ROS was induced in the small intestine, resulting in progressive mitochondrial dysfunction [14]. Decreased mitochondrial membrane potential and suppressed mitochondrial antioxidant defense system have been well documented in the small intestine challenged by LPS [15, 16]. Also, studies showed that the activity of the mitochondrial respiratory chain could be inactivated in response to the LPS challenge [17]. Cao et al. showed that LPS induced mitochondrial dysfunction, including the decreased membrane potential of intestinal mitochondria, intestinal content of mtDNA, and activities of the intestinal mitochondrial respiratory chain in piglets [16]. Fast-growing broiler chickens are more sensitive to LPS challenge at an early stage, which can be a promising alternative model for the research of dietary intervention that positively regulates performance and mitochondrial function in poultry. Recently, Sun and colleagues reported the beneficial effects of quercetin in a broiler chicken model of LPS-induced intestinal oxidative stress and mitochondrial dysfunction [18].

Curcuminoids generally consist of three major compounds: curcumin (60–70%), demethoxycurcumin (20–27%), and bisdemethoxycurcumin (10–15%) [19, 20]. Curcumin has been shown to possess mitochondria-protective properties in various animal models, including the LPS-induced animal model [21, 22]. Our previous studies showed that curcumin prevented the overproduction of ROS and attenuated the decrease of mitochondrial membrane potential in both D-galactosamine/LPS–treated mice and heat-stressed broilers [23, 24]. Recently, more attention has been drawn to developing the other two curcuminoids due to the low bioavailability and poor aqueous solubility of curcumin. Bisdemethoxycurcumin is a minor but not least important constituent of curcuminoids. Bisdemethoxycurcumin is chemically more stable than pure curcumin in physiological media and exhibits better bioavailability [25, 26]. It was reported that bisdemethoxycurcumin counteracted oxidant stress in mice by increasing GSH and superoxide dismutase (SOD) activities and alleviated cisplatin-induced renal injury by reducing the generation of ROS in renal tubular epithelial cells [27–29]. There is no clear supremacy of curcumin over bisdemethoxycurcumin in different models, whereas in some cases, bisdemethoxycurcumin shows more antioxidant potency for chemoprevention [30, 31]. Moreover, the pharmacological modulation of mitochondrial function following bisdemethoxycurcumin supplementation is predicted to be a critical mechanism for the prevention or therapy of disease.

The present study, therefore, is aimed at investigating the protective effect of bisdemethoxycurcumin on the mitochondrial dysfunction of jejunum and ileum in LPS-treated broilers. Not only did we examine the mitochondrial morphology, ROS production, mitochondrial membrane potential, mitochondrial redox system, and mitochondrial respiratory complex activity but we also discussed the transcriptional regulation of the mitochondrial antioxidant system and mitochondrial biogenesis by bisdemethoxycurcumin treatment.

## 2. Materials and Methods

### 2.1. Ethics Statement

The experimental protocol in the present study was approved by Nanjing Agricultural University Institutional Animal Care and Use Committee, China, and conducted in accordance with the Guidelines for Experimental Animals of the Ministry of Science and Technology (Beijing, P.R. China).

### 2.2. Experimental Design, Animals, and Diets

A total of 320 one-day-old Arbor Acres male broilers were allotted to one of four treatments with 8 replicates per treatment and 10 birds per replication. Treatments were arranged in a 2 × 2 factorial arrangement with main effects of diet (0 or 150 mg/kg bisdemethoxycurcumin supplementation) and stress (saline or LPS challenge) as follows: (1) broilers fed a basal diet (CON), (2) broilers fed a basal diet with 150 mg/kg bisdemethoxycurcumin (BIS), (3) broilers fed a basal diet with the challenge of LPS (LPS), and (4) broilers fed a basal diet with 150 mg/kg bisdemethoxycurcumin and LPS challenge (L-BIS) for a 20-day trial. Bisdemethoxycurcumin is provided by Kehu Bio-technology Research Center (Guangzhou, People's Republic of China.) with 98% purity. LPS is purchased from Sigma-Aldrich Chemical Co. (St. Louis, MO, USA) and from *Escherichia coli* serotype O55: B5. The doses of bisdemethoxycurcumin and LPS were according to our previous study, respectively [27, 32]. On days 16, 18, and 20 of the trial, broilers received an intraperitoneal injection of LPS (1 mL/kg body weight in sterile saline) in the LPS and L-BIS group while the same injection of sterile saline in the the CON and BIS group.

During the whole experimental period, broilers were housed in meshed floor of cages (120 × 70 × 60 cm), which were placed in vertical three-layer earth. Birds had ad libitum access to mash feed in a long feed trough and water by a nipple drinker, respectively. The basal diet (corn-soybean meal) is shown in Table 1 and is formulated to meet nutrient requirements of broilers (NRC, 1994). The room temperature was set at 32-34°C for days 0 to 14 and gradually decreased by 2 to 3°C per week to a final temperature of 25 ± 1°C. Broilers were provided with a 12L:12D light program throughout the experiment.

### 2.3. Sample Collection

Two hours after injection of LPS on days 20 of the trial, one bird was randomly selected from each replicate (*n* = 8) and killed by exsanguination. Sections of up to 1 cm in length were cut off from the middle of jejunum and ileum. The jejunal and ileal sections were gently flushed with ice-cold saline for the removal of digesta and then fixed in 2.5% ice-cold glutaraldehyde for examination of mitochondrial morphology. The mucosa of jejunum and ileum was scrapped by a glass slide and immediately stored in liquid nitrogen for further measurement.

### 2.4. Transmission Electron Microscopy

The fresh sliced jejunum and ileum tissues were fixed in a 2.5% glutaraldehyde solution (pH = 7.4, 0.1 mol/L sodium cacodylate buffer) and then postfixed 1% osmium tetroxide (v:v). The samples were dehydrated in series of ethanol and acetone concentrations and embedded in epoxy resin. The ultrastructure of jejunum and ileum was examined by transmission electron microscope (Hitachi H-7650).

### 2.5. Determination of Reactive Oxygen Species (ROS) and Mitochondrial Membrane Potential (MMP)

ROS production of jejunum and ileum was measured a commercial ROS assay kit (Beyotime Institute of Biotechnology, Haimen, China) by using 2,7-dichlorofluorescein diacetate as fluorescence probe according to the detailed describe of Zhang et al. [33]. The fluorescence was measured at the excitation wavelength of 488 nm and emission wavelength of 525 nm through a fluorescence spectrometer. Intracellular ROS production was determined by the mean DCFH-DA fluorescence intensity and presented as the percentage of the control group taken as 100%.

Mitochondrial membrane potential was measured using a commercial assay kit (Beyotime Institute of Biotechnology, Haimen, China) by using a Rhodamine 123 probe according to the detailed describe of Zhang et al. [33]. The fluorescence was measured at the excitation wavelength of 507 nm and emission wavelength of 529 nm through a fluorescence spectrometer. The results were determined by the mean fluorescence intensity and expressed as the percentage of the control group taken as 100%.

### 2.6. Measurements of Adenosine Triphosphate (ATP) Level

The ATP levels of jejunum and ileum were measured using an ATP content assay kit (Solarbio, Beijing, China) according to the manufacturer's instructions.

### 2.7. Preparation of Mitochondria

The jejunal and ileal mitochondria were isolated as previously described [21]. Briefly, the jejunal and ileal segments were separated and washed twice to get rid of contents. Then, the minced samples were homogenized in an ice-cold isolation buffer containing 10 mM Trizma hydrochloride, 250 mM sucrose, and 1 mM EDTA adjusted by Tris to pH 7.4 and centrifuged at 800 g for 5 min at 4°C. The supernatant was collected and centrifuged at 12,000 g for 15 min at 4°C. Thereafter, the obtained pellet was washed and spinned twice and finally resuspended in the ice-cold isolation buffer. Aliquots of mitochondrial suspension were stored at −80°C for the subsequent analysis.

### 2.8. Measurement of Mitochondrial Antioxidant Enzyme Activity and Metabolite Content

Mitochondrial GSH concentration, manganese superoxide dismutase (MnSOD) activity, and malondialdehyde (MDA) concentration were measured using commercial kits purchased from Nanjing Jiancheng (Nanjing, China) [34]. The results of GSH concentration, MnSOD activity, and MDA concentration were corrected by the protein concentrations and expressed as milligram per gram protein (mg/g protein), unit per milligram protein (U/mg protein), and nanomole per milligram protein (nmol/mg protein), respectively.

### 2.9. Quantitative Real Time-PCR and mtDNA Copy Number Analysis

Total RNA was isolated from homogenate tissues using TRIzol Reagent (Takara, Dalian, China). The RNA concentration was measured by the spectroscopy method using a NanoDrop ND-1000UV spectrophotometer (NanoDrop Technologies, Thermo Scientific, USA). The equal amounts of RNA were adopted and reverse transcribed to cDNA using a PrimeScript RT Reagent kit (Takara Biotechnology Co., Dalian, China). The resulting cDNA was used to measure the genes expression levels using the SYBR® Premix Ex Taq™ Kit (Takara Biotechnology Co. Ltd., Dalian, China) on the QuantStudio®5 real-time PCR Design & Analysis system (Applied Biosystems, USA). The PCR amplification reaction conditions consist of a prerun at 95°C for 30 s, 40 cycles of denaturation at 95°C for 5 s, and annealing at 60°C for 30 s. Each sample was run in duplicate, and melting curve analysis was performed to confirm the specificity of the reaction. The primer sequences of MnSOD, nuclear factor erythroid-2-related factor 2 (Nrf2), Trx2, TrxR2, Prx3, sirtuin-1 (SIRT1), PGC-1*α*, NRF2, NRF1, TRAM, and *β*-action are shown in Table 2. The relative expression levels of target genes were normalized with *β*-action and then calculated using the 2^−*ΔΔ*Ct^ method. The results were expressed as the percentage of the control group taken as 100%.

The mtDNA copy number was quantified using quantitative real time-PCR as previously described [35]. Briefly, total DNA of jejunum and ileum samples was extracted using the universal Genomic DNA Extraction Kit (TakaRa Biotechnology Co., Dalian, China). Aliquots of DNA were amplified and quantified by real-time PCR analysis as stated above. The abundance of mtDNA was normalized to that of *β*-actin and then calculated using the 2^−*ΔΔ*Ct^ method. The mtDNA displacement loop (D-loop) and *β*-Actin were used as mtDNA and nuclear DNA markers, respectively. The primers for mtD-loop and *β*-Actin are shown in Table 2.

### 2.10. Measurement of Mitochondrial Respiratory Complex Activities

The activities of mitochondrial respiratory complexes I-V were determined with commercial kits obtained purchased from SinoBestBio (Shanghai, China) according to the manufacturer's directions.

### 2.11. Statistical Analysis

Data was analyzed by two-way ANOVA using the general linear model procedure of SPSS 17.0 (SPSS Inc., Chicago, USA). The models for analysis of variables concern the responses of diet (0 or 150 mg/kg bisdemethoxycurcumin supplementation), stress (saline or LPS challenge), and their interaction as fixed effects in a 2 × 2 factorial arrangement. When the interaction showed significant differences, data was performed using one-way analysis of variance followed by Tukey's posthoc test for multiple comparisons. Data represent mean with their pooled standard errors and is considered significant at *P* < 0.05.

## 3. Results

### 3.1. Mitochondrial Ultrastructure

**Figure 1:**
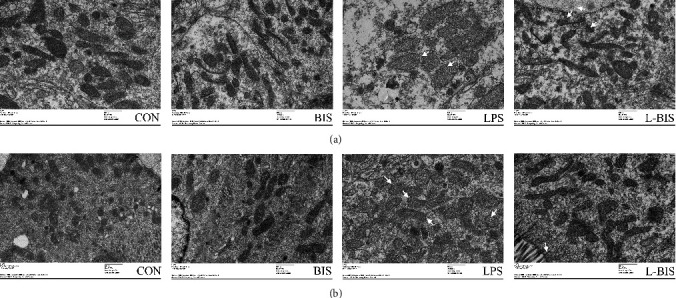
Effect of bisdemethoxycurcumin on mitochondrial ultrastructure of jejunum and ileum (a, b) in LPS-treated broilers. Arrows indicate mitochondrial deformed membrane and dilated cristae in the transmission electron microscopy images (original magnification: ×8000). CON: broiler chickens fed a basal diet; BIS: broiler chickens fed a basal diet with 150 mg/kg bisdemethoxycurcumin; LPS: broiler chickens fed a basal diet with LPS injection; L-BIS: broiler chickens fed a basal diet with LPS injection and 150 mg/kg bisdemethoxycurcumin.

As shown in Figure 1, the jejunal and ileal mitochondrial ultrastructure appeared largely unchanged in the BIS group versus the CON group. However, broilers challenged by LPS showed abnormal alterations in architecture of the jejunal and ileal mitochondria, characterized by more swollen mitochondria, elongated or enlarged cristae, and a lower matrix density as compared with the CON group. The severely damaged mitochondrial architecture in jejunum and ileum was partially attenuated by dietary bisdemethoxycurcumin treatment in broilers.

### 3.2. ROS Production and Mitochondrial Membrane Potential

**Figure 2:**
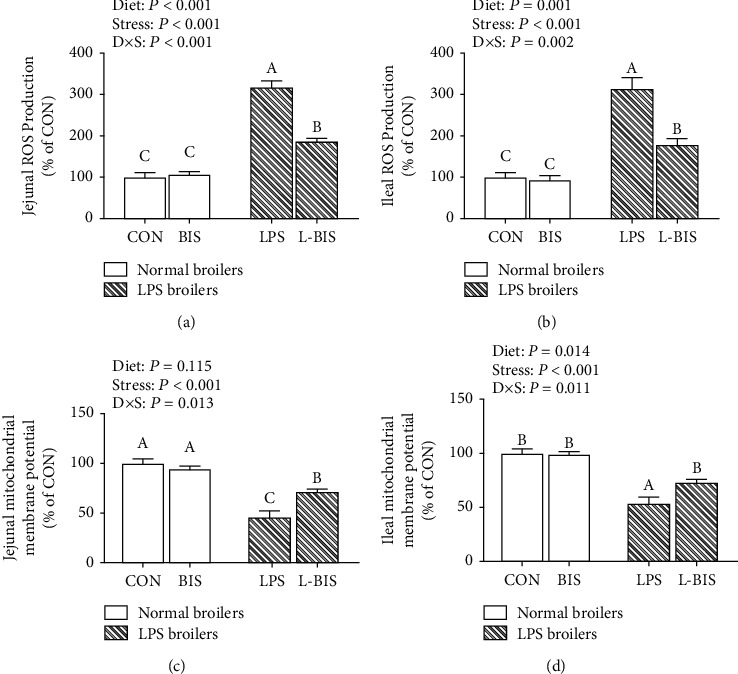
Effects of bisdemethoxycurcumin on the ROS production (a, b) and mitochondrial membrane potential (c, d) of jejunum and ileum in LPS-treated broilers. CON: broiler chickens fed a basal diet; BIS: broiler chickens fed a basal diet with 150 mg/kg bisdemethoxycurcumin; LPS: broiler chickens fed a basal diet with LPS injection; L-BIS: broiler chickens fed a basal diet with LPS injection and 150 mg/kg bisdemethoxycurcumin. Diet: 0 or 150 mg/kg bisdemethoxycurcumin treatment; stress: saline or LPS challenge; *D* × *S*: interaction between bisdemethoxycurcumin treatment and LPS challenge. Values represent the means ± SE and *n* = 6. ^A-C^ indicate the difference among groups is statistically significant (*P* < 0.05).

LPS challenge increased the jejunal and ileal ROS production (*P* < 0.05). Bisdemethoxycurcumin supplementation decreased the ROS production of jejunum and ileum (*P* < 0.05) (Figures 2(a) and 2(b)). There was an interaction between bisdemethoxycurcumin treatment and LPS challenge on the jejunal and ileal ROS production (*P* < 0.05). Compared to the LPS group, broilers in the L-BIS group showed lower ROS concentrations of jejunum and ileum (*P* < 0.05).

LPS challenge reduced the mitochondrial membrane potential of jejunum and ileum (*P* < 0.05) (Figures 2(c) and 2(d)). Bisdemethoxycurcumin supplementation increased the jejunal and ileal mitochondrial membrane potential (*P* < 0.05). There were interactions between bisdemethoxycurcumin treatment and LPS challenge on the mitochondrial membrane potential of jejunum and ileum (*P* < 0.05). Compared to the LPS group, broilers showed a higher mitochondrial membrane potential of jejunum and ileum in the L-BIS group (*P* < 0.05).

### 3.3. mtDNA Copy Number and ATP Levels

**Figure 3:**
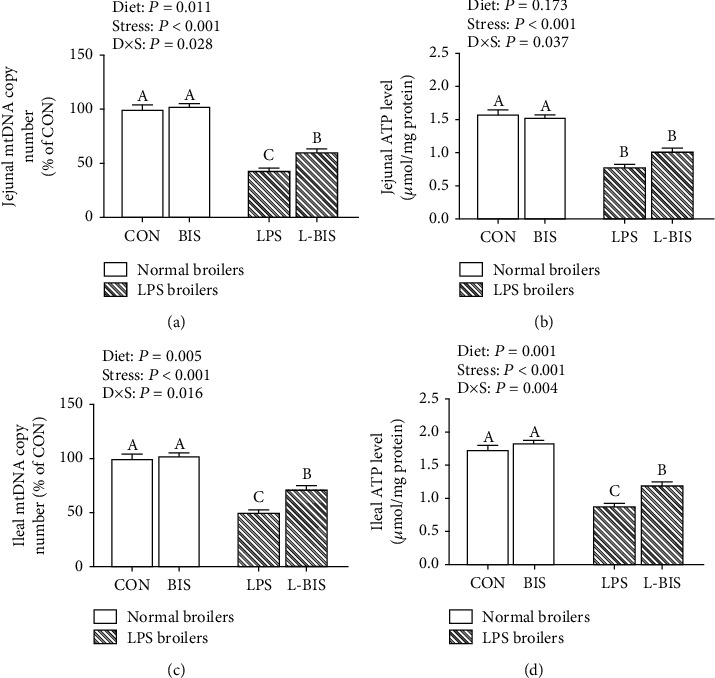
Effects of bisdemethoxycurcumin on the mtDNA copy number (a, c) and ATP levels (b, d) of jejunum and ileum in LPS-treated broilers. CON: broiler chickens fed a basal diet; BIS: broiler chickens fed a basal diet with 150 mg/kg bisdemethoxycurcumin; LPS: broiler chickens fed a basal diet with LPS injection; L-BIS: broiler chickens fed a basal diet with LPS injection and 150 mg/kg bisdemethoxycurcumin. Diet: 0 or 150 mg/kg bisdemethoxycurcumin treatment; stress, saline or LPS challenge; *D* × *S*: interaction between bisdemethoxycurcumin treatment and LPS challenge. Values represent the means ± SE and *n* = 8. ^A-C^ indicate the difference among groups is statistically significant (*P* < 0.05).

LPS challenge decreased the mtDNA copy number and ATP levels of jejunum and ileum (*P* < 0.05) (Figure 3). Bisdemethoxycurcumin supplementation increased the jejunal and ileal mtDNA copy number and ileal ATP levels (*P* < 0.05). There were interactions between bisdemethoxycurcumin treatment and LPS challenge on the mtDNA copy number and ATP levels of jejunum and ileum (*P* < 0.05). Compared to the LPS group, broilers showed higher jejunal and ileal mtDNA copy numbers and ileal ATP levels in the L-BIS group (*P* < 0.05).

### 3.4. Mitochondrial Redox System

LPS challenge decreased the GSH concentration and MnSOD activity, whereas increased MDA concentration of jejunum and ileum (*P* < 0.05) (Table 3). Bisdemethoxycurcumin supplementation increased the jejunal MnSOD activity, ileal GSH concentration, and MnSOD activity (*P* < 0.05). The ileal MDA concentration was decreased by bisdemethoxycurcumin supplementation (*P* < 0.05). The interactions between bisdemethoxycurcumin treatment and LPS challenge were observed on the jejunal MDA concentration, ileal MDA, GSH concentrations, and MnSOD activity (*P* < 0.05). Broilers in the L-BIS group had a lower ileal MDA concentration and a higher MnSOD activity as compared to the LPS group (*P* < 0.05).

### 3.5. The mRNA Expression of Mitochondrial and Cellular Antioxidant Genes

**Figure 4:**
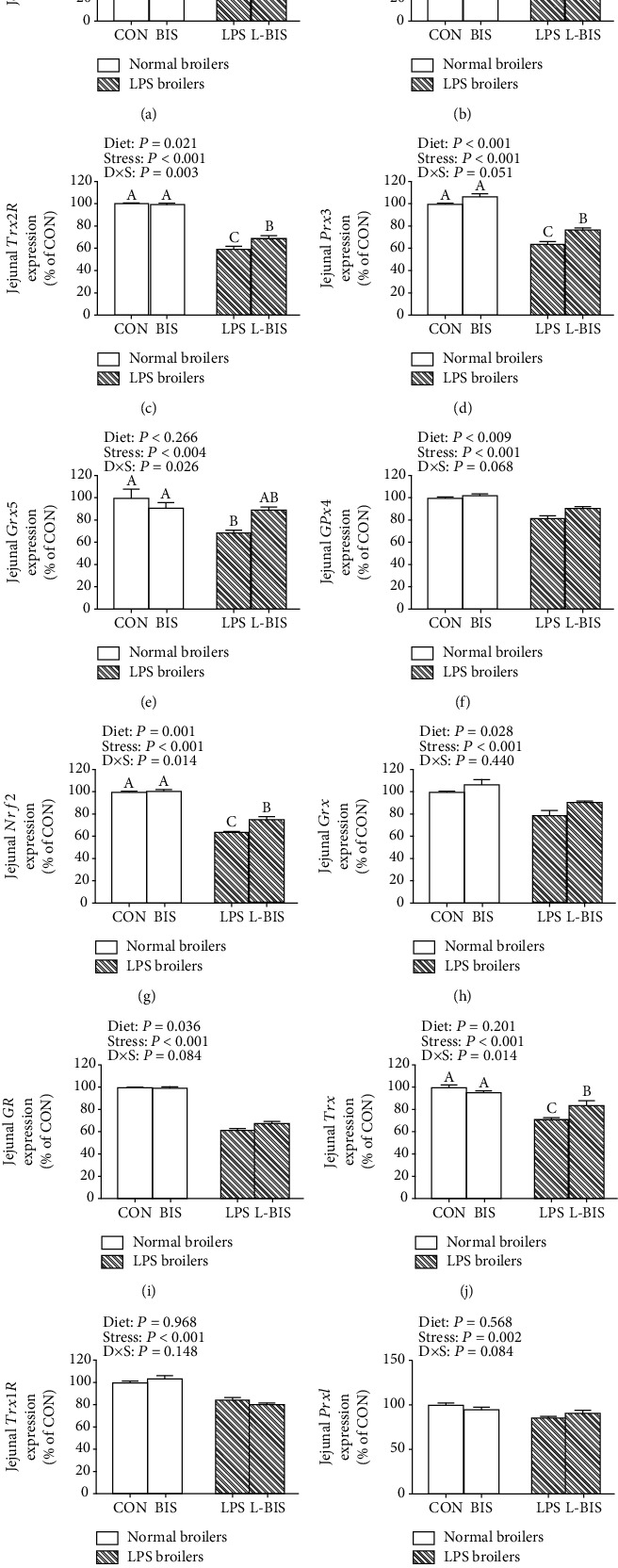
Effects of bisdemethoxycurcumin on the expression of mitochondrial and cellular antioxidant genes (a)–(l) of jejunum in LPS-treated broilers. MnSOD: manganese superoxide dismutase; Nrf2: nuclear factor erythroid-2-related factor 2; Prx3: peroxiredoxin-3; Trx2: thioredoxin 2; TrxR2: thioredoxin reductase 2. CON: broiler chickens fed a basal diet; BIS: broiler chickens fed a basal diet with 150 mg/kg bisdemethoxycurcumin; LPS: broiler chickens fed a basal diet with LPS injection; L-BIS: broiler chickens fed a basal diet with LPS injection and 150 mg/kg bisdemethoxycurcumin. Diet: 0 or 150 mg/kg bisdemethoxycurcumin treatment; stress: saline or LPS challenge; *D* × *S*: interaction between bisdemethoxycurcumin treatment and LPS challenge. Values represent the means ± SE and *n* = 8. ^A-C^ indicate the difference among groups is statistically significant (*P* < 0.05).

**Figure 5:**
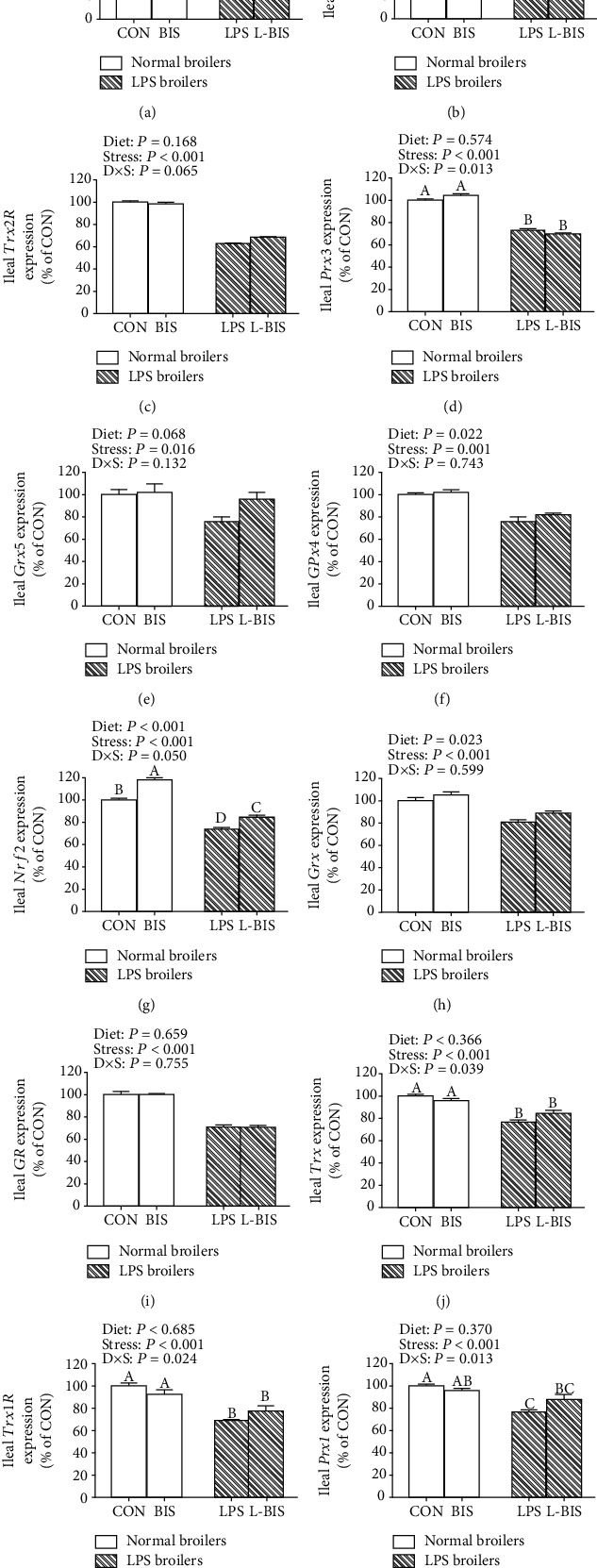
Effects of bisdemethoxycurcumin on the expression of mitochondrial and cellular antioxidant genes (a)–(l) of ileum in LPS-treated broilers. MnSOD: manganese superoxide dismutase; Nrf2: nuclear factor erythroid-2-related factor 2; Prx3: peroxiredoxin-3; Trx2: thioredoxin 2; TrxR2: thioredoxin reductase 2. CON: broiler chickens fed a basal diet; BIS: broiler chickens fed a basal diet with 150 mg/kg bisdemethoxycurcumin; LPS: broiler chickens fed a basal diet with LPS injection; L-BIS: broiler chickens fed a basal diet with LPS injection and 150 mg/kg bisdemethoxycurcumin. Diet: 0 or 150 mg/kg bisdemethoxycurcumin treatment; stress: saline or LPS challenge; *D* × *S*: interaction between bisdemethoxycurcumin treatment and LPS challenge. Values represent the means ± SE and *n* = 8. ^A-D^ indicate the difference among groups is statistically significant (*P* < 0.05).

LPS challenge decreased the mRNA expression levels of mitochondrial MnSOD, Trx2, TrxR2, Prx3, glutaredoxin5 (Grx5), glutathione peroxidase4 (GPx4), and cellular Nrf2, Grx, glutathione reductase (GR), Trx, thioredoxin 1 reductase (Trx1R), and Prx1 in jejunum and ileum (*P* < 0.05) (Figures 4 and 5). Bisdemethoxycurcumin supplementation increased the jejunal mRNA expression levels of MnSOD, TrxR2, Prx3, GPx4, Nrf2, Grx, and GR (*P* < 0.05). Bisdemethoxycurcumin supplementation increased the mRNA expression level of GPx4, Nrf2, and Grx in ileum (*P* < 0.05). There were interactions between bisdemethoxycurcumin treatment and LPS challenge on the jejunal mRNA expression levels of MnSOD, Trx2, TrxR2, Prx3, Grx5, Nrf2, and Trx (*P* < 0.05). Compared with the LPS group, bisdemethoxycurcumin supplementation increased the jejunal mRNA expression levels of MnSOD, Trx2, TrxR2, Prx3, Nrf2, and Trx in the L-BIS group (*P* < 0.05). There were interactions between bisdemethoxycurcumin treatment and LPS challenge on the ileal mRNA expression levels of TrxR2, Prx3, Nrf2, Trx, Trx1R, and Prx1 (*P* < 0.05). Compared with the LPS group, broilers showed an increased ileal mRNA expression level of Nrf2 in the L-BIS group (*P* < 0.05). Bisdemethoxycurcumin supplementation elevated the ileal mRNA expression level of Nrf2 in the BIS group as compared to the CON group (*P* < 0.05).

### 3.6. Mitochondrial Respiratory Complexes Activities

LPS challenge decreased the activities of mitochondrial respiratory complexes I-V in jejunum and ileum (*P* < 0.05) (Table 4). Bisdemethoxycurcumin supplementation increased the activities of mitochondrial respiratory complexes I, IV, and V in jejunum (*P* < 0.05). Bisdemethoxycurcumin supplementation increased the activities of mitochondrial respiratory complexes I-V in ileum (*P* < 0.05). There were interactions between bisdemethoxycurcumin treatment and LPS challenge on the activities of jejunal mitochondrial respiratory complexes I, II, V, and ileal mitochondrial respiratory complexes III and IV (*P* < 0.05). The activities of jejunal mitochondrial respiratory complexes I, II, and V were increased in the L-BUN group as compared to the LPS group (*P* < 0.05). Compared to the LPS group, broilers showed increased activities of ileal mitochondrial respiratory complexes III and IV in the L-BIS group (*P* < 0.05).

### 3.7. The mRNA Expression of Mitochondrial Biogenesis-Related Genes

**Figure 6:**
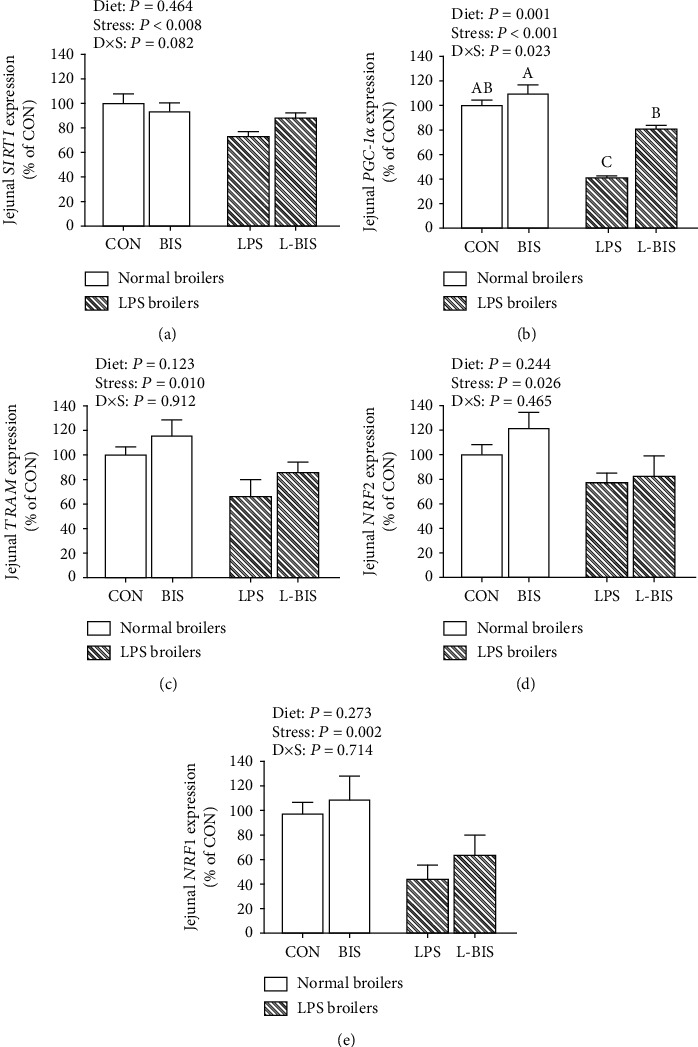
Effects of bisdemethoxycurcumin on the expression of mitochondrial biogenesis–related genes (a)–(e) of jejunum in LPS-treated broilers. NRF1: nuclear respiratory factor 1; NRF2: nuclear respiratory factor 2; PGC-1*α*: peroxisome proliferator activated receptor *γ* coactivator 1*α*; SIRT1: sirtuin-1; TFAM: mitochondrial transcription factor A. CON: broiler chickens fed a basal diet; BIS: broiler chickens fed a basal diet with 150 mg/kg bisdemethoxycurcumin; LPS: broiler chickens fed a basal diet with LPS injection; L-BIS: broiler chickens fed a basal diet with LPS injection and 150 mg/kg bisdemethoxycurcumin. Diet: 0 or 150 mg/kg bisdemethoxycurcumin treatment; stress: saline or LPS challenge; *D* × *S*, interaction between bisdemethoxycurcumin treatment and LPS challenge. Values represent the means ± SE and *n* = 8. ^A-C^ indicate the difference among groups is statistically significant (*P* < 0.05).

**Figure 7:**
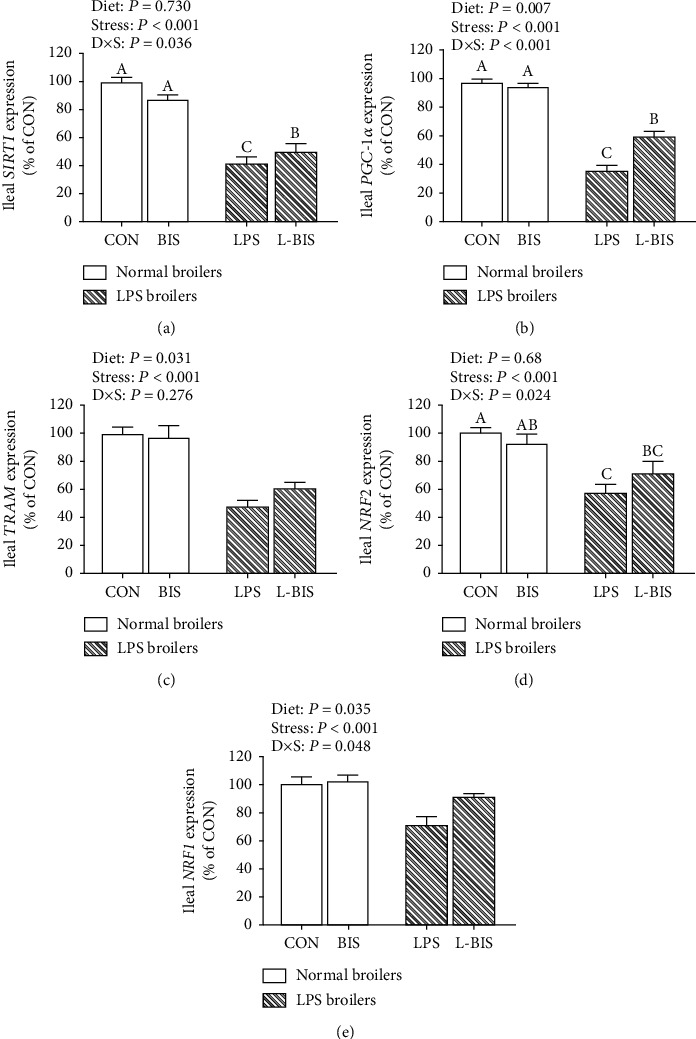
Effects of bisdemethoxycurcumin on the expression of mitochondrial biogenesis–related genes (a)–(e) of ileum in LPS-treated broilers. Effects of bisdemethoxycurcumin on the expression of mitochondrial biogenesis–related genes (a)–(e) of jejunum and ileum in LPS-treated broilers. NRF1: nuclear respiratory factor 1; NRF2: nuclear respiratory factor 2; PGC-1*α*: peroxisome proliferator activated receptor *γ* coactivator 1*α*; SIRT1: sirtuin-1; TFAM:mitochondrial transcription factor A. CON: broiler chickens fed a basal diet; BIS: broiler chickens fed a basal diet with 150 mg/kg bisdemethoxycurcumin; LPS: broiler chickens fed a basal diet with LPS injection; L-BIS: broiler chickens fed a basal diet with LPS injection and 150 mg/kg bisdemethoxycurcumin. Diet: 0 or 150 mg/kg bisdemethoxycurcumin treatment; stress: saline or LPS challenge; *D* × *S*: interaction between bisdemethoxycurcumin treatment and LPS challenge. Values represent the means ± SE, and *n* = 8. ^A-C^ indicate the difference among groups is statistically significant (*P* < 0.05).

LPS challenge decreased the mRNA expression of SIRT1, PGC-1*α*, NRF2, NRF1, and TFAM in jejunum and ileum (*P* < 0.05) (Figures 6 and 7). Bisdemethoxycurcumin supplementation increased the mRNA expression level of PGC-1*α* (*P* < 0.05) in jejunum and the mRNA expression levels of PGC-1*α*, NRF1, and TFAM in ileum (*P* < 0.05). The interactions between bisdemethoxycurcumin treatment and LPS challenge significantly were observed on the mRNA expression levels of PGC-1*α* in jejunum and ileum and the mRNA expression levels of NRF2 and NRF1 in ileum (*P* < 0.05). Compared to the LPS group, broilers showed higher mRNA expression levels of PGC-1*α* in jejunum and ileum (*P* < 0.05).

## 4. Discussion

Increasing evidence highlights mitochondrial dysfunction as one of the deleterious consequences in LPS-induced animal models. A dietary intervention is encouraged to prevent and mitigate LPS-induced mitochondrial dysfunction. In the present study, we demonstrated a natural product, bisdemethoxycurcumin, as a promising candidate to prevent small intestinal mitochondrial dysfunction and further elucidated the underlying mechanism in LPS-treated broilers partly. Our results suggested that the mitochondrial antioxidant system induced by bisdemethoxycurcumin contributed to the elimination of ROS, prevention of lipid peroxidation, and maintenance of redox balance within mitochondria. Moreover, our data showed that upregulation of the PGC-1*α* expression augmented mitochondrial biogenesis and promoted new mitochondria production by bisdemethoxycurcumin treatment, in accordance with the result of the increased mtDNA copy number we observed.

We found that bisdemethoxycurcumin could exert a beneficial effect in the preservation of mitochondrial morphology and simultaneously contribute to inhibition of ROS production and suppression of mitochondrial membrane depolarization. Mitochondrial morphology and mass are mechanistically linked to mitochondrial dysfunction and organ failure [36]. Impaired mitochondrial morphology has been implicated in increased electron leak from the mitochondrial respiratory chain and loss of mitochondrial membrane potential, which is the initial stage of mitochondrial dysfunction [37]. Bisdemethoxycurcumin has been widely used to suppress the burst of ROS and counteract oxidant stress in various diseases [26, 28, 38]. In our study, increased ROS levels and decreased mitochondrial membrane potential induced by LPS were partially rectified by dietary bisdemethoxycurcumin treatment. Interestingly, bisdemethoxycurcumin treatment also effectively increased the mtDNA copy number and ATP levels in LPS-treated broilers, indicating that the protective effect of bisdemethoxycurcumin is probably due to the augmented mitochondrial mass and number.

Redox homeostasis is increasingly implicated as a key event in the mitochondrial faction and the nuclear fraction. It has been hypothesized that LPS-induced intestinal toxicity and oxidant damage are associated with an imbalance of redox homeostasis within mitochondria. Mitochondria are the primary sources of both physiological and pathological ROS. Mitochondria are also important sites of ROS elimination and play an essential role in maintaining redox homeostasis. MnSOD exclusively localizes in the mitochondrial matrix and functions as a mitochondrial antioxidant enzyme that converts superoxide radicals into H_2_O_2_ and molecular oxygen. A previous study has shown similar induction of SOD activity by curcumin and bisdemethoxycurcumin in vivo [32]. In agreement with these findings, our results showed observable increases in mitochondrial MnSOD activity in jejunum and ileum, indicating its effects on redox homeostasis. In addition, bisdemethoxycurcumin treatment induced an increasing trend in GSH level and a significant decrease in MDA content, one of the best investigated lipid peroxidation byproducts, compared to the LPS group. In general, increased MnSOD enzyme activity induced by bisdemethoxycurcumin could accelerate the degradation of excessive ROS, manifested by the decreased ROS and MDA contents, thereby attenuating cell and mitochondrial oxidant damage.

The GSH-Grx and Trx systems are two important thiol-dependent antioxidant systems. These two redox systems work in parallel to synergistically maintain redox balance in cells [39]. The GSH-Grx system, consisting of GSH, GR, GPx, and Grx, specifically reduces protein-glutathione mixed disulfides [40]. In chicken, Grx is present in the cytosol and nucleus while Grx5 and GPx4 are in the mitochondrion [6]. Our results showed that LPS challenge decreased the mRNA expression of cytosolic and mitochondrial GSH-Grx and Trx systems, which would be mediated by the direct attacks of excessive ROS and indirect regulation of Nrf2 inactivation following LPS stimulation. In LPS-treated broiler, the mRNA expression of jejunal Grx5 was significantly increased by the supplementation of bisdemethoxycurcumin. This may be due to the highly increased GSH concentrations induced by bisdemethoxycurcumin via the direct free radical scavenging activity and promotion of GSH-GSSG turnover [27, 35]. One of the protective mechanisms for the active role of the mitochondrial Grx system is associated with keeping mitochondrial complex I functional by reversible oxidation-reduction reaction [41, 42]. Consistently, with the improved mitochondrial Grx system, dietary bisdemethoxycurcumin supplementation increased the mitochondrial complex I of Jejunum.

Furthermore, bisdemethoxycurcumin induced the upregulation of Trx2 and Prx3 in LPS-treated broiler, suggesting the activation of the mitochondrial Trx2 system by dietary bisdemethoxycurcumin administration. Mitochondria contain a variety of thioredoxin systems that are essential for redox control [43]. Among them, the Trx2 system, comprising Trx2, TrxR2, and Prx3, is specifically identified in mitochondria and functions in ROS elimination and protection against oxidative stress [44, 45]. Trx2, a redox-active protein, acts as an electron donor to reduce Prx3 and is simultaneously converted to its oxidized form. The oxidized Trx2 is then reduced back to Trx2 by NADPH-dependent TrxR2. This recycle accelerates the detoxification of ROS and peroxide, thereby attenuating the oxidant damage induced by ROS accumulation [8, 46]. A previous study from our laboratory confirmed the positive regulation of curcumin on the mitochondrial Trx2 system, which was mediated by the Nrf2 expression in broilers [35]. Trx2 is one of the antioxidant-related genes of the Nrf2 pathway and is activated by Nrf2 transcription [47, 48]. Our data also demonstrated the activation of Nrf2 induced by dietary bisdemethoxycurcumin administration in LPS-treated broilers. Therefore, we speculated that the protective effects of bisdemethoxycurcumin were mediated by Nrf2 activation and subsequent upregulation of the Trx2 system to maintain redox homeostasis within mitochondria. Moreover, our data showed an increased mRNA expression of MnSOD in jejunum following bisdemethoxycurcumin treatment in LPS-treated broilers. Trx could induce the expression of MnSOD [49, 50]. It is believed that the MnSOD-inducing property by Trx plays a critical role in protection against cell and mitochondrial damage [50]. MnSOD is also an antioxidant-related gene regulated by Nrf2, which could be induced by bisdemethoxycurcumin [27, 51]. However, it is not clear whether the induction of MnSOD by bisdemethoxycurcumin is mediated directly by Nrf2 activation or indirectly by Trx2 induction or is even involved in the interaction between Nrf2 and Trx2.

To validate that the decline of ATP levels and increase of ROS concentrations might be due to the disruption of the mitochondrial respiratory chain, we investigated the complex I-V activities in jejunum and ileum following the LPS challenge and bisdemethoxycurcumin treatment. Here, we demonstrated decreased complex I and II activities of jejunum in LPS-treated broilers which were prevented by dietary bisdemethoxycurcumin administration. In ileum, the complex III activity was increased by bisdemethoxycurcumin, but consistent increases of complex I and II activities were not observed. Complex I and III are the main sites of ROS production in mitochondria [52]. LPS injection impaired the enzyme activities of complexes I and III, leading to a burst of ROS from the mitochondrial respiratory chain [53, 54]. The accumulation of ROS is beyond the scavenging capacity of the intracellular antioxidant system and, in turn, exacerbates mitochondrial dysfunction, including compromised mitochondrial respiratory complex activities. It is assumed that the enhancement of the redox defense system and its scavenging activity by bisdemethoxycurcumin is a causal link to the decreased ROS, which counteracts oxidant damage within mitochondria to some extent. The induction of multiple antioxidant pathways by bisdemethoxycurcumin has been suggested as another possible explanation for the observed improvement of complex I and III activities in our study.

Besides the discrepant alterations of complex I-III activities by bisdemethoxycurcumin treatment in different intestinal segments, we found significant increases in complex IV activities in both jejunum and ileum. Complex IV catalyzes the terminal step of the mitochondrial electron transfer chain that reduces dioxygen to water and creates a proton gradient across the inner mitochondrial membrane [55]. Complex IV serves as one of the major regulation sites for oxidative phosphorylation but is not a critical site of ROS production [56]. The abnormal changes of complex IV activity are correlated with the ATP levels because ATP is synthesized via complex V in a proton gradient-dependent manner. We speculated that the regulation of bisdemethoxycurcumin on mitochondrial respiratory complex IV activity might be a compensatory mechanism to enhance the ATP synthesis. Efficient ATP production is essential for mitophagy and the removal of damaged mitochondria [57]. Concordantly, we reported increases in ATP contents in jejunum and ileum following bisdemethoxycurcumin treatment, especially a statistically significant increase in ileum. This might lead to inhibiting excessive ROS leak and further normalizing ROS production.

It will not be surprising that bisdemethoxycurcumin treatment might trigger the activation of the PGC-1*α* pathway in LPS-treated broilers. PGC-1*α* is a master regulator of mitochondrial biogenesis. PGC-1*α* activates and governs the transcriptional control of NRF1 and NRF2 and subsequently TFAM [11, 13]. This PGC-1*α* pathway plays a central role in the synthesis of mitochondrial DNA and proteins and the generation of new mitochondria [58, 59]. Our results showed that bisdemethoxycurcumin treatment significantly increased the mRNA expression of PGC-1*α* and NRF2. There is a strong connection between the activation of PGC-1*α* and mitochondrial biogenesis, which might result changes in mtDNA contents [60, 61]. In the present study, bisdemethoxycurcumin treatment consistently increased the mtDNA copy number of jejunum and ileum in LPS-treated broilers. This consistency between PGC-1 expression and mtDNA copy number confirmed our speculation that the beneficial effects of bisdemethoxycurcumin to attenuate mitochondrial dysfunction are linked to a mitochondrial biogenesis response involving the production of new mitochondria and replication of mtDNA. On the other hand, the promoters of NRF2, TFAM, and PGC-1*α* contain an antioxidant responsive element consensus sequence for the binding of Nrf2, indicating a possible way to stimulate mitochondrial biogenesis and mediate the synthesis of new mitochondria by Nrf2 activation [62, 63]. Moreover, early findings identified a positive feedback loop between Nrf2 and PGC-1*α* that promotes the nuclear localization of Nrf2 for activation of the antioxidant defense system, repairing oxidant damage and maintaining mitochondrial function [64, 65]. Considering that the upregulation of Nrf2 by bisdemethoxycurcumin was also observed in the present study, it was conceivable that the overlapping functions in the antioxidant defense system and synergistic actions in mitochondrial biogenesis of PGC-1*α* and Nrf2 might get involved in the regulation of mitochondria function and repair of mitochondrial oxidant damages by bisdemethoxycurcumin in LPS-treated broilers.

## 5. Conclusions

In summary, our results demonstrated that dietary bisdemethoxycurcumin supplementation at 150 mg/kg improved the mitochondrial antioxidant system, inhibited ROS production, and augmented mitochondrial biogenesis of jejunum and ileum in LPS-treated broilers. This might be associated with the activation of the mitochondrial antioxidant system and induction of the PGC-1*α* pathway by bisdemethoxycurcumin supplementation.

## Figures and Tables

**Table 1:** Formulation and calculated composition of the basal diet.

Period	1-20 d
Ingredient (%)	
Corn	57.00
Soybean meal (44.2%, crude protein)	31.30
Corn gluten meal (60%, crude protein)	3.90
Soybean oil	3.10
Dicalcium phosphate	1.80
Limestone	1.30
L-Lysine·	0.15
DL-Methionine	0.15
Premix	1.00
Salt	0.30
Total	100.00
Calculation of nutrients	
Metabolizable energy, kcal/kg	3033
Crude protein, %	21.52
L-Lysine, %	1.14
Methionine, %	0.50
Calcium, %	1.00
Total phosphorus, %	0.65
Available phosphorus, %	0.46
Arginine, %	1.36
Methionine+cystine, %	0.85

Provided per kg of diet: vitamin A (trans-retinyl acetate), 10,000 IU; vitamin D3 (cholecalciferol), 3,000 IU; vitamin E (all rac-*α*-tocopherol acetate), 30 IU; menadione, 1.3 mg; thiamin, 2.2 mg; riboflavin, 8 mg; nicotinamide, 40 mg; choline chloride, 600 mg; calcium pantothenate, 10 mg; pyridoxine·HCl, 4 mg; biotin, 0.04 mg; folic acid, 1 mg; vitamin B12 (cobalamin), 0.013 mg; Fe (from ferrous sulfate), 80 mg; Cu (from copper sulfate), 8 mg; Mn (from manganese sulfate), 110 mg; Zn (from zinc oxide), 65 mg; I (from calcium iodate), 1.1 mg; and Se (from sodium selenite), 0.3 mg.

**Table 2:** Primer sequences of targeted genes and *β-*actin.

Gene	GeneBank ID	Primer sequence (5′ →3′)	Product size (bp)
*β*-Actin	NM_205518.1	TGCTGTGTTCCCATCTATCG	150
		TTGGTGACAATACCGTGTTCA	
mtD-loop	XM_015291451.1	AGGACTACGGCTTGAAAAGC	198
		CATCTTGGCATCTTCAGTGCC	
GPx 4	NM_001346449	TTACGTGATGCTCCCCTTCG	176
		AATCTTCGGGTCTGCCTCAC	
GR	XM_040671422.1	TCCTGACTACGGCTTCGAGA	150
		AACTTGCCGTAACCACGGAT	
Grx	NM_205160.1	CCGTCCCTCGTGTGTTTATT	107
		CACCAGAGCACCAATTTGAC	
Grx 5	NM_001008472.1	CTGGCCTACCATCCCACAAG	128
		GAAGTGCTGAGCGGATTCCT	
MnSOD	NM_204211.1	AGGAGGGGAGCCTAAAGGAGA	214
		CCAGCAATGGAATGAGACCTG	
NRF1	NM_001030646.1	AAGAACACGGCGTGACTCAA	274
		TCGCTTCCGTTTCTTACCCG	
NRF2	NM_001007858.1	GAGCCCATGGCCTTTCCTAT	212
		CACAGAGGCCCTGACTCAAA	
Nrf2	NM_205117.1	GATGTCACCCTGCCCTTAG	215
		CTGCCACCATGTTATTCC	
PGC-1*α*	AB170013.1	GACGTATCGCCTTCTTGCTC	157
		CTCGATCGGGAATATGGAGA	
Prx1	NM_001271932.1	AGCTGTAATGCCAGATGGGC	137
		CAGCTCTGTCACTGTACGCA	
Prx3	XM_004942320.1	ACCTCGTGCTCTTCTTCTACC	110
		ACCACCTCGCAGTTCACATC	
SIRT1	NM_001004767.1	CGCAGCCCGATAACTTCCTT	206
		CGTTTCTGGGAGCAGGTCTT	
TFAM	NM_204100.1	GTGAAAGCCTGGCGAAACTG	228
		CACAGCTCAGGTTACACCGT	
Trx	NM_205453.1	GGTGAAGAGCGTGGGCAATC	99
		GGTCCACACCATGTGGCAGA	
Trx2	NM_001031410.1	AGTACGAGGTGTCAGCAGTG	141
		CACACGTTGTGAGCAGGAAG	
Trx1R	NM_001352023.1	GCCAAGTCCACCAAGGATGA	170
		GGCACGTTTGTTTGCTCCAT	
Trx2R	NM_001122691.1	CCGGGTCCCTGACATCAAA	94
		TAGCTTCGCTGGCATCAACA	

GPx4: glutathione peroxidase4; GR: glutathione reductase; Grx: glutaredoxin; Grx5: glutaredoxin5; MnSOD: manganese superoxide dismutase; NRF1: nuclear respiratory factor 1; NRF2: nuclear respiratory factor 2; Nrf2: nuclear factor erythroid-2-related factor 2; PGC-1*α*: peroxisome proliferatoractivated receptor *γ* coactivator 1*α*; Prx1: peroxiredoxin 1; Prx3: peroxiredoxin-3; SIRT1: sirtuin-1; TFAM: mitochondrial transcription factor A; Trx: thioredoxin; Trx2: thioredoxin 2; Trx1R: thioredoxin 1 reductase; Trx2R: thioredoxin 2 reductase.

**Table 3:** Effects of bisdemethoxycurcumin on the mitochondrial redox system of jejunum and ileum in LPS-treated broilers.

Items	LPS (-)		LPS (+)		SEM	*P*		
	CON	BIS	LPS	L-BIS		Diet	Stress	*D* × *S*
Jejunal mitochondria								
MDA (nmol/mg protein)	2.61^c^	3.14^bc^	5.75^a^	4.33^ab^	0.216	0.313	<0.001	0.032
GSH (mg/g protein)	11.45^a^	12.42^a^	7.50^b^	6.69^b^	0.219	0.859	<0.001	0.053
MnSOD (U/mg protein)	20.56	23.07	14.87	17.56	0.227	<0.001	<0.001	0.845
Ileal mitochondria								
MDA (nmol/mg protein)	2.20^c^	2.19^c^	6.28^a^	4.87^b^	0.165	0.042	<0.001	0.044
GSH (mg/g protein)	11.67^a^	11.10^a^	7.15^b^	8.85^b^	0.257	0.283	<0.001	0.036
MnSOD (U/mg protein)	21.01^a^	21.49^a^	16.00^b^	18.79^c^	0.179	<0.001	<0.001	0.003

MDA: malondialdehyde; GSH: glutathione; MnSOD: superoxide dismutase. CON: broiler chickens fed a basal diet; BIS: broiler chickens fed a basal diet with 150 mg/kg bisdemethoxycurcumin; LPS: broiler chickens fed a basal diet with LPS injection; L-BIS: broiler chickens fed a basal diet with LPS injection and 150 mg/kg bisdemethoxycurcumin. Diet: 0 or 150 mg/kg bisdemethoxycurcumin treatment; Stress: saline or LPS challenge; *D* × *S*: interaction between bisdemethoxycurcumin treatment and LPS challenge. Data were expressed as means and SEM, *n* = 8 replicates per treatment and 10 birds per replication. Means within a row with no common superscript differ *P* < 0.05.

**Table 4:** Effects of bisdemethoxycurcumin on the activities of the mitochondrial respiratory complexes (I-V) of jejunum and ileum in LPS-treated broilers.

Items	LPS (-)		LPS (+)		SEM	*P*		
	CON ^2^	BIS	LPS	L-BIS		Diet	Stress	*D* × *S*
Jejunum								
Complex I (*μ*mol NADH/min/mg protein)	9.90^a^	9.85^a^	6.16^b^	8.83^a^	0.237	0.014	<0.001	0.011
Complex II (*μ*mol NADH/min/mg protein)	6.76^a^	6.64^a^	4.46^b^	6.28^a^	0.215	0.066	0.007	0.038
Complex III (*μ*mol cytochrome *c*/min/mg protein)	16.66	17.12	11.51	14.54	0.528	0.118	0.002	0.242
Complex IV (*μ*mol CoQH_2_/min/mg protein)	28.98^a^	28.49^a^	17.12^b^	24.19^a^	0.633	0.019	<0.001	0.009
Complex V (*μ*mol DCPIP/min/mg protein)	44.95	47.27	32.62	42.05	1.266	0.034	0.003	0.180
Ileum								
Complex I (*μ*mol NADH/min/mg protein)	10.12	9.98	6.87	8.19	0.132	0.042	<0.001	0.130
Complex II (*μ*mol NADH/min/mg protein)	6.29	6.52	4.29	5.85	0.142	0.006	<0.001	0.320
Complex III (*μ*mol cytochrome *c*/min/mg protein)	20.00^a^	19.27^a^	12.96^b^	18.59^a^	0.412	0.009	<0.001	0.001
Complex IV (*μ*mol CoQH_2_/min/mg protein)	27.56^ab^	28.10^a^	20.87^c^	24.61^b^	0.374	0.011	<0.001	0.048
Complex V (*μ*mol DCPIP/min/mg protein)	42.23	44.76	28.36	36.84	0.733	0.002	<0.001	0.059

CON: broiler chickens fed a basal diet; BIS: broiler chickens fed a basal diet with 150 mg/kg bisdemethoxycurcumin; LPS: broiler chickens fed a basal diet with LPS injection; L-BIS: broiler chickens fed a basal diet with LPS injection and 150 mg/kg bisdemethoxycurcumin. Diet: 0 or 150 mg/kg bisdemethoxycurcumin treatment; stress: saline or LPS challenge; *D* × *S*: interaction between bisdemethoxycurcumin treatment and LPS challenge. Data were expressed as means and SEM, *n* = 8 replicates per treatment and 10 birds per replication. Means within a row with no common superscript differ *P* < 0.05.

## Data Availability

The data used to support the findings of this study are available from the corresponding author upon request.

## Abbreviations

ATP: Adenosine triphosphate
GPx: Glutathione peroxidase
GSH: Glutathione
GR: Glutathione reductase
Grx: Glutaredoxin
LPS: Lipopolysaccharide
MDA: Malondialdehyde
MMP: Mitochondrial membrane potential
MnSOD: Manganese superoxide dismutase
mtDNA: Mitochondrial DNA
Nrf2: Nuclear factor erythroid-2-related factor 2
NRF1: Nuclear respiratory factor 1
NRF2: Nuclear respiratory factor 2
PGC-1*α*: Peroxisome proliferator activated receptor *γ* coactivator 1*α*
Prx: Peroxiredoxin
ROS: Reactive oxygen species
SIRT1: Sirtuin-1
SOD: Superoxide dismutase
TFAM: Mitochondrial transcription factor a
Trx: Thioredoxin
TrxR: Thioredoxin reductase.
